# Feasibility and Long-Term Efficacy of PEComa Treatment—20 Years of Experience

**DOI:** 10.3390/jcm10102200

**Published:** 2021-05-19

**Authors:** Aleksandra Sobiborowicz, Tomasz Świtaj, Paweł Teterycz, Mateusz J. Spałek, Anna Szumera-Ciećkiewicz, Michał Wągrodzki, Marcin Zdzienicki, Anna M. Czarnecka, Piotr Rutkowski

**Affiliations:** 1 Department of Soft Tissue/Bone, Sarcoma and Melanoma, Maria Sklodowska-Curie National Research Institute of Oncology, 02-781 Warsaw, Poland; ola.sob96@gmail.com (A.S.); Tomasz.Switaj@pib-nio.pl (T.Ś.); Pawel.Teterycz@pib-nio.pl (P.T.); Mateusz.Spalek@pib-nio.pl (M.J.S.); Marcin.Zdzienicki@coi.pl (M.Z.); Piotr.Rutkowski@pib-nio.pl (P.R.); 2 Medical Faculty, Medical University of Warsaw, 02-091 Warsaw, Poland; 3 Departament of Computional Oncology, Maria Sklodowska-Curie National Research Institute of Oncology, 02-781 Warsaw, Poland; 4 Department of Pathology and Laboratory Diagnostics, Maria Skłodowska-Curie Institute—Oncology Center, 02-781 Warsaw, Poland or szumann@gmail.com (A.S.-C.); michal.wagrodzki@pathologist.cc (M.W.); 5 Department of Diagnostic Hematology, Institute of Hematology and Transfusion Medicine, 00-791 Warsaw, Poland

**Keywords:** perivascular epithelioid cell tumor, PEComa, angiomyolipoma, lymphangioleiomyomatosis, sarcoma

## Abstract

Perivascular epithelioid cell tumors (PEComas) represent a family of rare mesenchymal neoplasms, some of which are malignant. There are no specific management guidelines for PEComas, and factors correlating with the disease course are not well defined. This analysis aimed to describe the outcomes of PEComa patients treated radically, including those treated exclusively in the national reference sarcoma center. The secondary aim of the study was to analyze factors associated with PEComa treatment efficacy. We performed an analysis of 27 patients subsequently treated radically for PEComa between 1999 and 2019 who were in follow-up in the national sarcoma reference center. The proportional-hazards model was used to compare the risk of death. The median age at diagnosis was 45 (21–67) years, and 67% of patients were female. The median follow-up period was 68 months (95% CI: 39–101). At the time of analysis, eleven patients (40.7%) experienced progression of the disease and four (14.8%) died. Surgery in the reference sarcoma center was associated with a longer disease control (log-rank *p* < 0.001). The 5-year-OS rate was 88% (95% CI: 74–100) for the whole analyzed group. We concluded that PEComa treatment should be managed in reference sarcoma centers by a multidisciplinary tumor board with an experienced surgical team. Microscopically radical resection is associated with a longer disease-free survival. Patients requiring long-term follow-ups as late recurrence may be expected.

## 1. Introduction

Perivascular epithelioid cell tumors (PEComas) represent a family of mesenchymal tumors developing from perivascular epithelioid cells (PECs) [1]. The World Health Organization (Geneva, Switzerland) defines PEComa as unusual mesenchymal tumors composed of histologically and immunohistochemically distinctive perivascular epithelioid cells. The term PEComa was introduced in 1996 to describe a family of lesions characterized by the presence of perivascular epithelioid cells [2]. The PEComa family includes renal and extrarenal angiomyolipomas (AMLs), a clear-cell sugar tumor (CCST) of the lung and primary extrapulmonary sugar tumor (PEST), lymphangioleiomyomatosis (LAM), a clear-cell myomelanocytic tumor (CCMT) of the falciform ligament/ligamentum teres, a primary cutaneous PEComa (CCCMT-cutaneous clear cell myomelanocytic tumor), and a PEComa NOS (not otherwise specified) [3]. This variety of neoplasms with different clinicopathological characteristics share a molecular origin, with frequent inactivating mutations in TSC1 (Tuberous Sclerosis Complex 1) and TSC2 (Tuberous Sclerosis Complex 2), resulting in hyperactivation of the mTOR signaling pathway [4,5].

The most common and well-described PEComas are renal AMLs—representing the majority of benign renal tumors [6]. Typically, a sporadic form of AML occurs as a small, well-circumcised, asymptomatic lesion with a high fatty tissue content, diagnosed accidentally during imaging studies performed due to unrelated indications [6]. AMLs associated with tuberous sclerosis often present as multiple, large lesions, which can lead to progressing renal failure and life-threatening retroperitoneal hemorrhages [7]. In a small percentage of AMLs (both TSC-associated and sporadic), the dominance of epithelioid, multinucleated cells is observed [8], known as epithelioid AMLs (EAMLs), which, in some cases, become malignant [9,10]. LAM most commonly affects women of child-bearing age [11]. The sporadic type of LAM affects around 1 in 400,000 women with no known predisposing factors, while TSC-associated LAM is diagnosed in 30–40% of female patients with TSC [12]. LAM is characterized by a multifocal proliferation of smooth muscle cells, PECs, and pathological lymphatic vessels, resulting in the progressive disruption of the lung parenchyma, development of numerous cysts, and chylous effusions, leading to pulmonary insufficiency [12,13]. Rarely, LAM tumors are an extrapulmonary cystic lesion comprising masses of abnormal lymphatic vessels filled with lymph, and are localized in mediastinal lymph nodes, the retroperitoneal space, or the pelvis [14]. These lesions pose a therapeutic challenge, as they are typically poorly demarcated and localized in close proximity to vital organs, which limits surgical management [15]. Finally, PEComa NOS is a collective term for tumors composed of PECs that do not classify into any of the aforementioned subtypes [1]. The boundary between malignant extrarenal EAMLs and PEComas NOS is difficult to determine [16]. PEComa NOS, like EAMLs, may be malignant, both in terms of local recurrences and the development of metastases [16] or local aggressive behavior [17,18].

Radical resection remains the primary treatment modality for PEComas, as they are characterized by resistance to radiation and chemotherapy [16,19]. Treatment modalities showing the potential effectiveness in the management of unresectable PEComas include hormonotherapy [20] and mTOR inhibitors [18]. In fact, management guidelines of these tumors have not been widely described. The aim of this study was to describe the outcomes of patients with PEComas treated in a reference national sarcoma center. The secondary aim of the study was to define potential factors associated with therapy efficacy. As malignant PEComas are extremely rare, with an estimated incidence of 0.12–0.24/1,000,000—that is 4 to 8 patients in Poland—our analysis is expected to cover the majority of the patients in the country.

## 2. Materials and Methods

### 2.1. Analyzed Group

We performed an analysis of consecutive PEComa patients who were in follow-up at the Maria Sklodowska-Curie National Research Institute of Oncology (MSCNRIO, Warszawa, Poland), including those diagnosed and treated for PEComas in our national reference sarcoma center. Our department in the MSCNRIO is the only sarcoma multidisciplinary treatment center in Poland, and it is, therefore, a national reference center. Patients included in the analysis started treatment between 1 January 1999 and 31 December 2019. Our follow-up data cut-off was 31 March 2021. Specific inclusion criteria were: (1) male or female patients ≥18 years of age, (2) histologically confirmed diagnosis of PEComa, (3) available formalin-fixed paraffin-embedded (FFPE) sample from core needle biopsy, (4) available CT scan at treatment start; (5) history of radical surgical treatment for localized PEComa. Patients with primarily unresectable and metastatic disease were excluded from the analysis. The histopathology of all enrolled patients was reviewed in our Institute by experienced sarcoma pathologists (A.S.-C. and M.W.). FFPE samples, 2.5 μm in thickness, were stained with hematoxylin and eosin, and primary antibodies were used for immunohistochemistry (Table S1) to confirm the histopathological diagnosis. Patients were treated as per national sarcoma treatment guidelines [21,22,23].

### 2.2. Analyzed Data

Electronic medical records of CGM CLININET HIS (CompuGroup Medical Poland Ltd., Lublin, Poland) were screened with MedStream Designer (MSD) software (Transition Technologies, Łódź, Poland). The corresponding 10th revision of the International Statistical Classification of Diseases and Related Health Problems (ICD) C48-C49 and the keyword ‘PEComa’ or ‘Perivascular epithelioid cell neoplasm’ were used. Data were reviewed independently by two researchers. Data on death were confirmed by the Polish National Cancer Registry at the Department of Epidemiology and Cancer Prevention (http://onkologia.org.pl/, accessed on 31 March 2021) via the personal identification number of the patients at 31 March 2021. The CONSORT flow diagram of patients enrolled was prepared with CONsolidated Standards of Reporting Trials on-line software (CONSORT Group, Canada).

### 2.3. Statistical Analysis

The continuous variables were summarized by the median and inter-quantile range (IQR), while categorical variables were summarized by the count and percentage of total cases. All point estimates were reported with a 95% confidence interval (CI) unless stated otherwise. The Kaplan–Meier estimator with the log-rank test, as well as the Cox proportional hazard model, were used for the survival analysis. The median follow-up time was calculated using the reverse Kaplan–Meier estimator. All analyses were performed in the R language environment version 3.6.3 (The R Foundation for Statistical Computing, Indianapolis, USA) with abundant use of tidyverse and survminer packages. The *p* ≤ 0.05 was deemed statistically significant.

## 3. Results

### 3.1. Treated Patients

**Figure 1:**
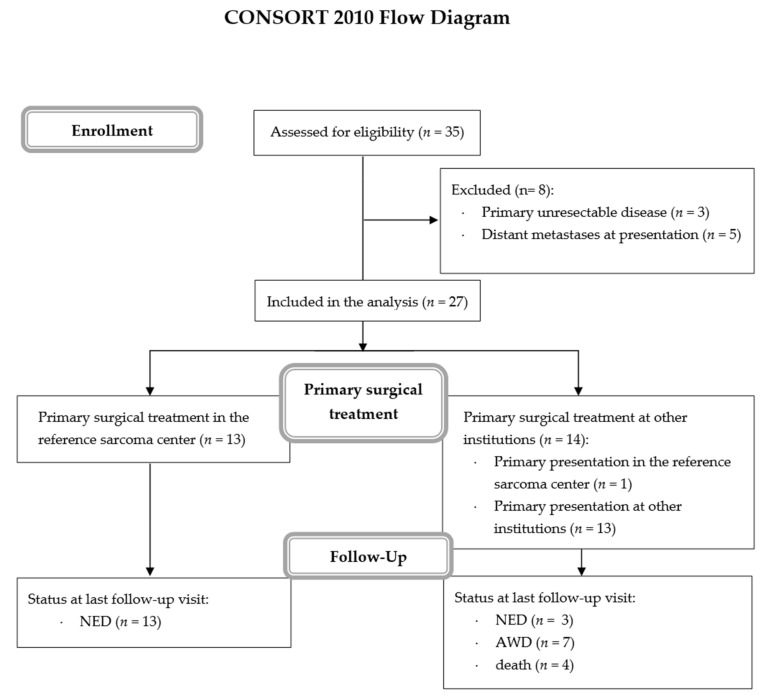
Treatment and follow-up of PEComa patients treated in Maria Sklodowska-Curie National Research Institute of Oncology.

First, 35 PEComa patients were treated in our center, including 27 patients treated radically (Figure 1). Of the remaining eight patients, three were diagnosed with primary unresectable extrapulmonary LAM and five were diagnosed with metastases at presentation, and all these cases were, thus, excluded from further analyses. Thirteen patients included in our analysis underwent primary surgical resection of PEComas in our center (Table S2), while 14 were diagnosed initially in regional institutions and were referred to the sarcoma center for follow-up and treatment after the first surgery (Table S3).

### 3.2. Clinical Presentation

**Figure 2:**
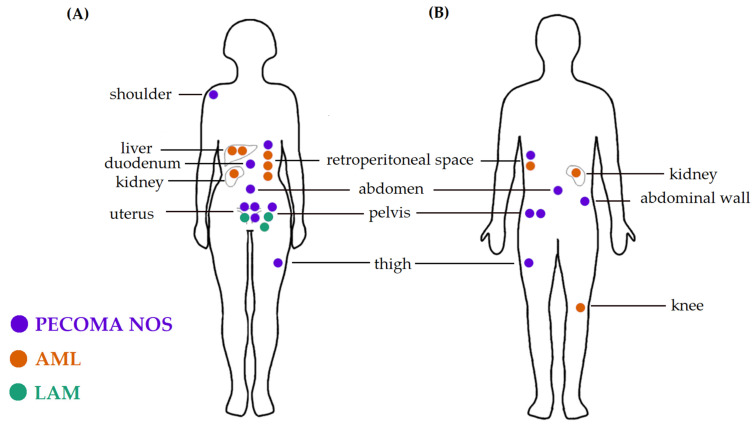
Graphical representation of anatomic localizations of primary tumors in female (**A**) and male (**B**) patients. Figure by Aleksandra Sobiborowicz.

PEComa NOS was the most common pathologic diagnosis (55.6% of patients), followed by epithelioid AML (33.3%) and LAM (11.1%). The median age at diagnosis in the whole group was 45 years (range: 21–67 years), for the PEComa NOS group—48 years (range: 23–67 years), epithelioid AML—45 years (range: 32–63 years), and for LAM—31 years (range: 21–38 years). Females accounted for the majority of patients (67%, 18/27), which was also true in a subpopulation of patients with PEComas localizing outside of the genitourinary tract (59.1%, 13/22). In PEComa NOS patients, the female-to-male ratio was 1.33:1, and in epithelioid AML, it was 2:1, while patients with LAM were solely female. The most common anatomical localizations of the primary tumors were: the retroperitoneal space (22.2%), the soft tissues of the pelvis (18.5%), and the uterus (14.8%) (Figure 2). The clinicopathological characteristics of patients treated primarily in our center and in other hospitals are summarized in Tables S1 and S2, accordingly.

### 3.3. Tumors Pathology

**Figure 3:**
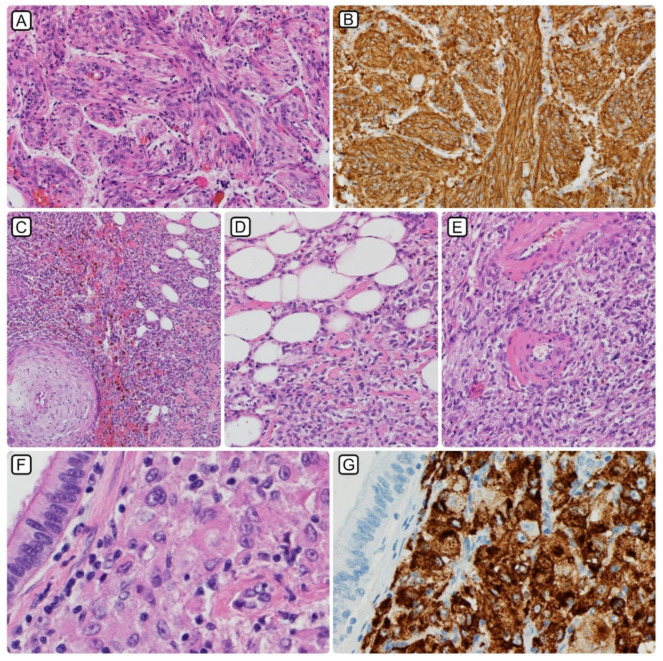
Histopathological spectrum of PEComa; retroperitoneal LAM: (**A**)—HE (200×); (**B**)—SMA (200×). AML: (**C**)—classical presentation with a mixture of spindle cell component, thick, hyalinizing vessels, and mature adipose tissue (100×), (**D**)—mature adipose tissue and fibrous stroma (200×), (**E**)—thick, hyalinizing vessels (200×); PEComa, NOS: (**F**)—PEComa metastasis to the lung, where the bronchial epithelium is seen in the upper left corner (400×), (**G**)—PEComa metastasis in HMB-45 immunostaining (400×).

All PEComas-NOS were of typical histological appearance with a biphasic growth pattern of spindle cells and epithelioid cells, arranged around vascular spaces. Immunohistochemically, the tumor cells were positive for melanocytic (HMB-45 and Melan-A/MART-1) and myoid (desmin, smooth muscle actin, and muscle-specific actin/all muscle actin/HHF-35) markers (Figure 3). The mean diameter of primary tumors (17 cases) was 6.9 cm (range: 1.4–21.0 cm), that of PEComas NOS was 6.1 cm (1.5–11.0 cm), that of epithelioid AMLs was 7.3 cm (1.4–21.0 cm), while that of primary LAM tumors reached 12 cm.

### 3.4. Surgical Treatment

Fourteen patients were referred to our clinic with primary, up-front resectable tumors, 13 of whom continued the diagnostic process and treatment in our clinic, while one underwent emergency resection at the regional institution due to major bleeding (Figure 1). Out of the remaining 13 patients, seven were referred to our center after macroscopically radical resection and without evidence of the disease progression, one patient was referred due to local relapse of the disease, two patients were referred due to local relapse and development of distant metastases, while three were referred solely due to the development of distant metastases. The clinical course of the disease of patients treated primarily in our center and in other institutions is summarized in Table 1 and Table 2, accordingly.

Outside of reference center, microscopically radical resection (R0) was achieved only in four patients; in seven cases, tumor infiltrate was detected in the surgical margin (R1); one case had the macroscopically residual disease (R2); for two cases, margins were not described (Rx). Of the 13 patients treated surgically in our center, for 11, R0 resection was achieved, and for two, R1 resection was achieved.

### 3.5. Radiation Therapy

**Figure 4:**
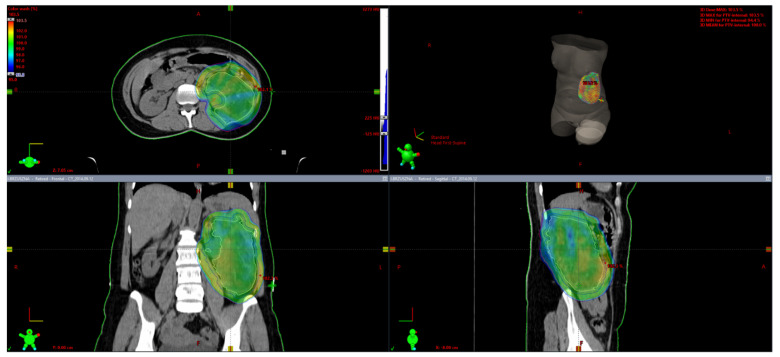
Radiotherapy treatment plan with dose distribution (50.4 Gy of preoperative radiotherapy in 28 fractions) for a patient with locally advanced retroperitoneal PEComa. Figure by Mateusz J. Spałek (Varian Aria Software).

Five patients received neoadjuvant radiotherapy with the intent to enable efficient surgery. The first and the second patient with locally advanced PEComas localized near the knee joint and the shoulder participated in our prospective study with preoperative 5 × 5 Gy radiotherapy with immediate surgery. The third patient with a marginally resectable PEComa of the knee joint and thigh was irradiated using a conventionally fractionated preoperative regimen, namely 2 to 50 Gy within 5 weeks. Two patients with locally advanced retroperitoneal tumors received 50.4 Gy of preoperative radiotherapy in 28 fractions (see treatment plan with dose distribution in Figure 4). We did not observe any significant toxicity of preoperative radiotherapy. Patients did not receive neoadjuvant or adjuvant chemotherapy.

### 3.6. Follow-Up

The median follow-up time was 68.5 months (95% CI: 39–101). The longest follow-up time reached 229 months. The last local relapse was noted after 132 months of observation, and the metastatic spread of the disease was noted after 107 months. At the time of analysis, 11 patients (40.7%) experienced progression of the disease—one patient (3.7%) developed local relapse only, five patients (18.5%) developed distant metastases only, and five patients developed both local relapse and distant metastases (18.5%). Four patients (14.8%) died during the time of observation. Four patients did not continue follow-up after the first year.

**Figure 5:**
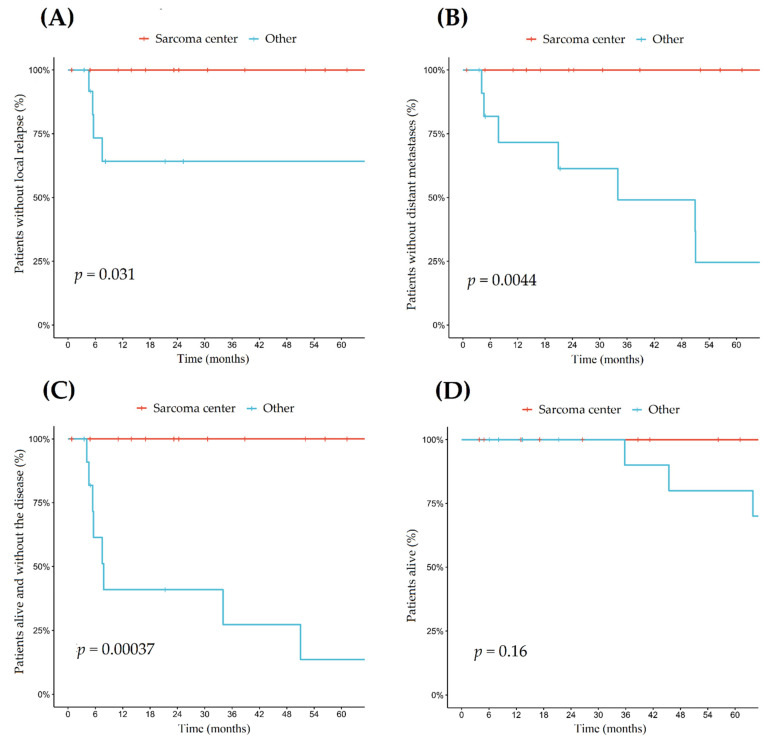
Survival analysis including: (**A**) 5—local recurrence-free survival; (**B**) 5—distant metastases-free survival, (**C**) 5—median disease-free survival, and (**D**) 2—overall survival.

All cases with local relapses were reported in patients who underwent the first resection of the primary tumor outside of the reference sarcoma center. There were no local relapses observed in patients operated in our institution, as the first surgery. In the case of resection performed in regional hospitals, there were four local relapses noted in the first year of observation (six in total). Therefore, the LRFS rate at 12 months reached 64% (95% CI: 41–99%). LRFS was stable in the long observation period at 48 months (Figure 5A).

Local relapse was most commonly observed in patients diagnosed with PEComa NOS (3/6). There were also two cases of local relapse noted in a patient with extrapulmonary LAM and one in the patient diagnosed with AML. In the analyzed cohort, there were no distant metastases noted in the case of patients treated surgically in our reference center (Figure 5B, Table S1).

The most common anatomic localization of metastases were the lungs (5/10) and the abdomen (5/10). Rare localizations of metastases included: the liver (1/10), pelvic lymph nodes (1/10), bones (1/10), and the retroperitoneal space (1/10). Six out of ten patients with distant metastases were diagnosed with PEComa NOS. Four patients with distant metastases (to the lungs, abdomen, and pelvic lymph nodes) underwent surgical resection of metastatic lesions, and in one of them, it allowed for complete disease control for 56 months of follow-up, without any additional treatment modalities.

### 3.7. Survival Efficacy

At the time of analysis, patients treated initially in our center did not experience disease relapse, progression, or death, and they were in follow-up with the time of observation between 12.8 and 52 months. For the patients who underwent surgery outside the national reference center, the median distant metastases-free survival (DMFS) was 34 months (7.8-NA) with a 2-year DMFS rate of 61% (95% CI: 38–99%). Surgery performed in the reference sarcoma center was associated with the longer local disease control (log-rank *p* < 0.005, Figure 5A). The median disease-free survival (DFS) in patients treated outside the reference sarcoma center was only 7.8 months (95% CI: 5.4-NA), with a 5-year DFS of 14% (95% CI: 2–80) with *p* < 0.01 for comparison with the sarcoma center cohort (Figure 5C), because, at the median time of observation of 41.3 months, patients treated initially in our center did not develop any local recurrence of metastases, and all remained alive. Those differences in the local control did not translate into a difference in the overall survival (OS) of the patients after a median of 4 years of observation. The 5-year OS rate was 88% (95% CI: 74–100) for the whole group (Figure 5D). Tumor size, its primary localization, or the patient’s sex or age statistically correlated with DFS, LRFS, and OS.

## 4. Discussion

The rarity and complexity of malignant PEComas pose significant challenges in interpreting its clinical and pathological characteristics, as well as drawing collective conclusions about the proper management of this entity. The largest review on this topic conducted by Bleeker et al., (2012) compiled 234 cases of PEComa NOS reported in the literature [16]. It is worth pointing out that this analysis included cases of extrarenal epithelioid AMLs—which, together with PEComa NOS, accounted for 85.7% of our cohort. The clinicopathological characteristics of cases included in our study remain in concordance with results of the aforementioned analysis, where the reported median age at diagnosis reached 43 years (range: 3–97 years), the mean tumor size was 6.8 cm, and a strong female predominance (73% of cases) was observed [16]. Similar clinical features were noted in several smaller case series [24,25], indicating that in all available reports, PEComas NOS generally affects middle-aged female patients; however, the range of the age at the diagnosis was wide, and both sexes can be affected. Some differences can be observed in the distribution of anatomic localization, as in our cohort, the most common sites of primary tumors were the retroperitoneal space, pelvis, and uterus, while, in the report by Bleeker et al., (2012), the skin and the liver/falciform ligament, together with the uterus and the retroperitoneal space, accounted for a substantial number of cases [16]. This may be attributed to the fact that PEComas of the falciform ligament tends to occur in the pediatric population, which was not represented in our cohort, while cutaneous PEComas are characterized by a benign course and can be easily managed with simple excision, so those patients may have been less likely to be referred to our reference center. The only effective curative treatment of PEComas is radical surgery with microscopically clear margins; however, multivisceral resections are rarely necessary [26,27].

Unfortunately, many patients with soft tissue sarcoma, including PEComa, may be subject to an initial suboptimal surgery, which happens more often if the multidisciplinary team (MDT) is not available for treatment planning. Suboptimal surgery often results in the subsequent need for more extensive surgery and/or radiotherapy, which would not be required if the primary tumor was initially assessed by an experienced team. In particular, wide re-excisions after an unplanned primary excision of STS often result in functional or cosmetic deficits [28]. In fact, the optimal management of STS relies on an appropriately performed core needle biopsy, accurate pathological diagnosis (Figure 3) and staging, followed by an effective surgical treatment and watchful surveillance after resection [29]. It has been proven that adherence to sarcoma treatment guidelines is important for patient survival but stricter in specialized reference sarcoma centers [29,30]. Moreover, it was also shown that between 30 and 45% of first histological diagnoses, provided by regional hospitals, are modified in sarcoma reference centers at expert reading. Moreover, in about 1 in 5 cases in the expert review, there was also a change in tumor grade in the sarcoma center. Such a diagnosis modification may also result in different therapeutic decisions. It should be remembered that expert opinion improves the quality of sarcoma diagnosis [31,32]. Finally, adherence to treatment guidelines for surgery was shown as the strongest independent prognostic factor of PFS in STS, along with age, gender, grade, and tumor size. Moreover, for OS again, adherence to treatment guidelines for surgery is an independent prognostic factor [33]. Therefore, care for sarcoma patients should be highly centralized [18,21,22,23,30].

The only effective curative treatment of PEComas is radical surgery with microscopically clear margins; however, multivisceral resections are rarely necessary [26,27]. Our analysis underlines the importance of the surgical management of those rare entities in specialized sarcoma centers. In our cohort, 14 patients underwent resection of the primary tumor in institutions not specialized in the management of soft tissue sarcomas, and only two of them had their tumor biopsied beforehand, which is not a relevant approach in sarcomas [34,35]. Among those 14 patients, one had local recurrence, five developed distant metastases, and five developed both. At the same time, in thirteen patients treated surgically in our center, no such events were reported. Primary treatment in the sarcoma center was associated with a significantly higher 2-year disease-free survival, which confirms the above-mentioned data from France or Canada. DFS was significantly longer in patients treated for the primary disease in our center when compared to patients treated in other facilities; however, this difference did not influence the overall survival, which did not differ between those two groups. A lack of OS difference may be attributed to the small sample size, but also to the fact that the survival of patients with progression of the disease tends not to be severely impaired. This is because, in our cohort, distant relapses could be managed by secondary surgeries, as in the case of our study where four patients with distant metastases (to the lungs, abdomen, and pelvic lymph nodes) underwent surgical resection of metastatic lesions. Moreover, there is growing evidence of the clinical responses of those tumors to several systemic therapies, such as mTOR and VEGFR inhibitors [36], which could also impact the survival of our patients.

Treatment outcomes of PEComas in retrospective analyses vary. In the review by Bleeker et al., (2012), out of 189 patients with follow-up data available, 38 (21.1%) had evidence of recurrence, and 20 (10.6%) of them died of the disease [16]. However, this analysis included patients treated in different institutions with different treatment approaches, and a large proportion of patients were treated for cutaneous PEComa, which harbors a favorable prognosis. In a pathological analysis of a case series by Doyle et al., (2013), 35 patients with PEComa NOS of the gastrointestinal tract had undergone surgical resection of the primary tumor, with 30 of the resections being macroscopically radical [24]. During a mean of 45 months of follow-up (range of 2–176 months), local recurrence was not observed in any of the patients; however, 13 patients (37.1%) developed distant metastases [24]. Folpe et al., (2005) performed such an analysis on 24 patients with PEComa NOS, and they noted three cases (13%) of local recurrence and five cases (21%) of distant metastasis during the mean 30 months of follow-up [25]. In all of the aforementioned reports and in concordance with our results, distant metastases occurred most commonly in the lungs and the abdomen, including the liver.

Folpe et al., (2005) proposed the stratification of PEComas NOS into three risk categories, based on the microscopic features of the tumors [25]. High-risk characteristics included: size >5 cm, infiltrative growth pattern, high nuclear grade, high cellularity, intratumor necrosis, vascular invasion, and mitotic rate ≥1/50HPF [25]. Bleeker et al., (2012) applied those criteria to analyzed cases and found that none of the tumors classified as benign relapsed, while out of the patients with progression of the disease after primary surgery, 81.6% were classified as high risk [16]. Pathologic features associated with a high risk of malignant clinical course for epithelioid AMLs include size >5 cm, infiltrative growth pattern, atypical epithelioid cells accounting for >50% of the tumor, vascular invasion, association with tuberous sclerosis, and in the case of renal epithelioid AMLs—an invasion of the renal vein [9,37,38]. In our analysis, tumor size, its primary localization, or the patient’s sex or age did not affect the DFS, LRFS, or OS in a statistically significant way, probably due to the small sample size typical for PEComa epidemiology.

Taken together, these data show that even though local or distant recurrence rates can vary, in most retrospective analyses, disease progression occurs in 20% to 50% of cases, underlining the importance of the careful management and long-term follow-up of these patients. Moreover, due to disease epidemiology, our report is the largest long-term single sarcoma center PEComa surgical treatment analysis, and only future multi-institutional analyses may provide additional information on optimal treatment approaches. In terms of the management of PEComas NOS and epithelioid AMLs, in the vast majority of cases, radical resection is also the only treatment method applied, as, in the report by Bleeker et al., (2012), only 18% of patients received other treatment modalities [16]. The reluctant use of neoadjuvant and adjuvant radio- and chemotherapy is attributed to the fact that PEComas are considered resistant to those treatments and, apart from several reports, there were no clear benefits of such an approach noted [39].

The limitations of this study include its retrospective character. Despite that, this study provides valuable data concerning the clinical management of PEComas and underlines the importance of their management in reference sarcoma centers, as well as the long-term follow-up of those patients.

## Figures and Tables

**Table 1:** The clinical course of the disease of patients treated surgically for PEComa exclusively at the reference sarcoma center.

Pt.	Sex	Age	Subtype	DM at Presentation	Preoperative RT	Surgical Margin	Developed LR	Developed DM	Death	Follow-Up Time (m)
1	F	54	AML	No	No	R0	No	No	No	41.3
2	F	48	AML	No	No	R0	No	No	No	38.7
3	F	33	NOS	No	Yes, 5 × 5 Gy	R0	No	No	No	26.5
4	M	32	NOS	No	No	R0	No	No	No	13
5	F	21	LAM	No	No	R0	No	No	No	100.5
6	F	48	NOS	No	No	R1	No	No	No	80.9
7	M	58	NOS	No	No	R1	No	No	No	126.7
8	F	39	AML	No	Yes, 28 × 1.8 Gy	R0	No	No	No	79.1
9	F	45	AML	No	Yes, 28 × 1.8 Gy	R0	No	No	No	61.1
10	M	23	NOS	No	No	R0	No	No	No	4.9
11	F	38	AML	No	No	R0	No	No	No	3.9
12	F	63	AML	No	No	R0	No	No	No	17.1
13	M	48	NOS	No	Yes, 5 × 5 Gy	R0	No	No	No	56.3

Abbreviations: DM—distant metastases; F—female; LR—local relapse; M—male; m—months; RPS—retroperitoneal space; RT—radiotherapy.

**Table 2:** The clinical course of the disease of patients treated surgically for PEComa outside of the reference sarcoma center.

Pt.	Sex	Age	Subtype	Surgical Margin	Status at Presentation at Our Center	LR Resection at Our Center	Preoperative RT	LR; Time to LR ^&^ (m)	DM; Time to DM ^&^ (m)	Death	Follow-Up Time (m)
1	F	33	NOS	R0	LR after multiple resections and DM	No	No	Yes; NA	Yes; NA	Yes	229.4
2	M	36	AML	R1	DM	No	No	No	Yes; 51	No	109
3	M	32	AML	R1	LR	Yes	Yes, 25 × 2 Gy	Yes; 5.4	Yes; 20.9	Yes	35.8
4	F	37	NOS	R1	Scar after resection	No	No	No	Yes; 7.8	No	13.3
5	F	48	NOS	R0	Scar after resection	No	No	No	No	No	8.1
6	F	39	NOS	R0	Scar after resection	No	No	No	No	No	6.1
7	M	61	NOS	ND	DM	No	No	No	Yes; 4.1	Yes	45.5
8	F	58	NOS	R1	Scar after resection	No	No	Yes; 5.6	No	No	81.9
9	M	67	NOS	R1	PT	No	No	No	No	No	21.3
10	F	47	NOS	R1	Scar after resection	No	No	No	Yes; 34	No	101.8
11	F	38	LAM	R0	Scar after resection	No	No	Yes; 131.5	Yes; 106.5	No	213
12	M	61	AML	R1	DM	No	No	No	Yes; 1.2	No	68.5
13	F	58	NOS	ND	Scar after resection	Yes	No	Yes; 7.5	Yes; 50.9	Yes	63.9
14	F	31	LAM	R2	LR and DM	No	No	Yes; 4.6	Yes; 4.6	No	71.5

Abbreviations: DM—distant metastases; F—female; LR—local relapse; M—male; m—months; NA—not applicable; ND—no data; PT—primary tumor; RPS—retroperitoneal space; RT—radiotherapy; ^&^ since the resection of the primary tumor.

## Data Availability

All data generated or analyzed during this study are available upon reasonable request upon DTA consent.

## Supplementary Materials

The following are available online at https://www.mdpi.com/article/10.3390/jcm10102200/s1, Table S1: A panel of primary antibodies used for immunohistochemistry to confirm the histopathological diagnosis of PEComa, Table S2: Clinicopathological characteristics of patients treated surgically for PEComa exclusively at the reference sarcoma center, Table S3: Clinicopathological characteristics of patients treated surgically for PEComa outside of the reference sarcoma center.
