# Supplementary material for: Feasibility and Long-Term Efficacy of PEComa Treatment—20 Years of Experience
Source: J Clin Med. 2021 May 19;10(10):2200. doi: 10.3390/jcm10102200 (PMC8160690; doi:10.3390/jcm10102200)
Supplement: Supplementary file 1 [file jcm-10-02200-s001.zip › jcm-1150044-supplementary.pdf]

Supplementary Materials

Table S1. A panel of primary antibodies used for immunohistochemistry to confirm the histopathological diagnosis of PEComas.

Primary antibody	Clone	Dilution	pH	Manufacturer/Distributor
**SMA**	1A4	RTU	9.0	Dako/Agilent
**Desmin**	D33	RTU	9.0	Dako/Agilent
**h-caldesmon**	h-CD	RTU	9.0	Dako/Agilent
**S100p**	POLI	RTU	9.0	Dako/Agilent
**SOX-10**	SP267	RTU	-	Roche/Ventana
**CD34**	QBEnd	RTU	9.0	Dako/Agilent
**ERG**	EP111	RTU	6.0	Dako/Agilent
**CKAE1/AE3**	CKAE1/AE3	RTU	9.0	Dako/Agilent
**Cathespin K**	3F9	1:50	6.0	Dako/Agilent
**HMB-45**	HMB-45	RTU	9.0	Dako/Agilent
**Melan A**	A103	RTU	9.0	Dako/Agilent
**MITF**	D5	1:50	9.0	Dako/Agilent
**TFE3**	MRQ37	RTU	-	Roche/Ventana
**CD163**	MRQ26	RTU	-	Roche/Ventana
**Ki-67**	MIB1	RTU	6.0	Dako/Agilent

Table S2. Clinicopathological characteristics of patients treated surgically for PEComa exclusively at our tertiary center

Pt.	Sex	Age	Diagnosis year	Subtype	Primary tumor site	Tumor size (cm)	Status at presentation	DM at presentation	Preoperative RT	Surgical margin	Developed LR	Developed DM	Death	Follow-up time (m)
**1**	F	54	2015	AML	RPS	6	Primary tumor without biopsy	No	No	R0	No	No	No	41.3
**2**	F	48	2016	AML	liver	4.5	Primary tumor without biopsy	No	No	R0	No	No	No	38.7
**3**	F	33	2017	NOS	shoulder	4.1	Primary tumor after biopsy	No	Yes. 5x5Gy	R0	No	No	No	26.5
**4**	M	32	2018	NOS	abdominal wall	4.2	Primary tumor without biopsy	No	No	R0	No	No	No	13
**5**	F	21	2010	LAM	pelvis	12	Primary tumor after biopsy	No	No	R0	No	No	No	100.5
**6**	F	48	2012	NOS	RPS	5	Primary tumor without biopsy	No	No	R1	No	No	No	80.9
**7**	M	58	2008	NOS	pelvis	11	Primary tumor without biopsy	No	No	R1	No	No	No	126.7

**8**	F	39	2014	AML	RPS	13.5	Primary tumor without biopsy	No	Yes. 28x1.8Gy	R0	No	No	No	79.1
**9**	F	45	2015	AML	RPS	21	Primary tumor without biopsy	No	Yes. 28x1.8Gy	R0	No	No	No	61.1
**10**	M	23	2018	NOS	pelvis	4.2	Primary tumor without biopsy	No	No	R0	No	No	No	4.9
**11**	F	38	2020	AML	liver	3	Primary tumor without biopsy	No	No	R0	No	No	No	3.9
**12**	F	63	2017	AML	kidney	1.4	Primary tumor without biopsy	No	No	R0	No	No	No	17.1
**13**	M	48	2014	NOS	thigh	4.9	Primary tumor after biopsy	No	Yes. 5x5Gy	R0	No	No	No	56.3

Abbreviations: DM – distant metastases. F- female; LR – local relapse. M-male; m – months; RPS – retroperitoneal space; RT - radiotherapy

Table S3. Clinicopathological characteristics of patients treated surgically for PEComa outside of our tertiary center

Pt.	Sex	Age	Diagnosis year	Biopsy before resection	Surgical margin	Subtype	Primary tumor site	Size(cm)	DM at diagnosis	Year of presentation at our center	Status at presentation at our center	LR resection at our center	Preoperative RT	DM; time to DM[&] (m)	LR; time to LR[&] (m)	Death	Follow-up time (m)
**1**	F	33	1998	No	R0	NOS	pelvis	ND	No	2013	LR after multiple resections and DM	No	No	Yes; ND	Yes; ND	Yes	229.4
**2**	M	36	2010	No	R1	AML	RPS	ND	No	2015	DM	No	No	Yes; 51	No	No	109
**3**	M	32	2014	Yes	R1	AML	knee	15	No	2015	LR	Yes	Yes. 25x2Gy	Yes; 20.9	Yes; 5.4	Yes	35.8
**4**	F	37	2017	No	R1	NOS	uterus	ND	No	2018	Scar after resection	No	No	Yes; 7.8	No	No	13.3
**5**	F	48	2018	No	R0	NOS	uterus	ND	No	2018	Scar after resection	No	No	No	No	No	8.1
**6**	F	39	2018	No	R0	NOS	thigh	ND	No	2018	Scar after resection	No	No	No	No	No	6.1
**7**	M	61	2012	No	ND	NOS	abdomen	ND	No	2012	DM	No	No	Yes; 4.1	No	Yes	45.5
**8**	F	58	2012	No	R1	NOS	duodenum	15	No	2013	Scar after resection	No	No	No	Yes; 5.6	No	81.9
**9**	M	67	2017	No	R1	NOS	RPS	90	No	2017	PT	No	No	No	No	No	21.3
**10**	F	47	2011	No	R1	NOS	uterus	ND	No	2011	Scar after resection	No	No	Yes; 34	No	No	101.8
**11**	F	38	2001	No	R0	LAM	uterus	ND	No	2012	Scar after resection	No	No	Yes; 106.5	Yes; 131.5	No	213

**12**	M	61	2014	No	R1	AML	kidney	ND	Yes	2014	DM	No	No	Yes; 1.2	No	No	68.5
**13**	F	58	2008	No	ND	NOS	abdome n	110	No	2008	Scar after resection	Yes	No	Yes; 50.9	Yes; 7.5	Yes	63.9
**14**	F	31	2013	No	R2	LAM	miednic a mniejsz a	ND	No	2014	LR and DM	No	No	Yes; 4.6	Yes; 4.6	No	71.5

Abbreviations: DM – distant metastases; F- female; LR – local relapse; M-male; m – months; NA- not applicable; ND – no data; RPS – retroperitoneal space; RT – radiotherapy; & since the resection of the primary tumour
